# Retrospective analysis of long-term gastrointestinal symptoms after *Clostridium difficile* infection in a nonelderly cohort

**DOI:** 10.1371/journal.pone.0209152

**Published:** 2018-12-17

**Authors:** Tamar F. Barlam, Rene Soria-Saucedo, Omid Ameli, Howard J. Cabral, Warren A. Kaplan, Lewis E. Kazis

**Affiliations:** 1 Section of Infectious Diseases, Department of Medicine, Boston University School of Medicine, Boston, Massachusetts, United States of America; 2 Department of Pharmaceutical Outcomes and Policy, College of Pharmacy at the University of Florida, Gainesville, Florida, United States of America; 3 Department of Health Law, Policy and Management, Boston University School of Public Health, Boston, Massachusetts, United States of America; 4 Department of Biostatistics, Boston University School of Public Health, Boston, Massachusetts, United States of America; 5 Center for Global Health and Development, Boston University School of Public Health, Boston, Massachusetts, United States of America; 6 Health Outcomes Unit, Department of Health Law, Policy and Management, Boston University School of Public Health, Boston, Massachusetts, United States of America; Cleveland Clinic, UNITED STATES

## Abstract

Elderly patients and those with comorbid conditions are at high risk for poor outcomes after *Clostridium difficile* infection (CDI) but outcomes in a healthier, nonelderly population are not well described. We sought to investigate gastrointestinal diagnoses and CDI during hospitalizations in the 24 to 36 months after an initial episode of CDI in nonelderly patients in a cohort with an overall low prevalence of comorbid conditions. We performed a retrospective analysis of hospital admissions from 2010–2013 using the Truven MarketScan database of employment-based private insurance claims. Subjects <65 years of age and their adult dependents (> = 18 years old); a CDI diagnosis in 2011 (index date); at least 12 months of pre-index continuous enrollment; and 24–36 months of continuous post-index enrollment were included. The 12 months of each subject’s enrollment prior to the index date for a CDI served as the reference period for the analyses of that subject’s post-CDI time periods. Hospital claims during the follow-up period were evaluated for gastrointestinal diagnoses and/or CDI ICD-9 codes. The risk of gastrointestinal diagnoses was assessed using Cox proportional hazards models adjusted for a pre-specified set of baseline demographic and clinical factors. During 2011, 5,632 subjects with CDI met the inclusion criteria for our study. The risk of gastrointestinal diagnoses in patients with a CDI diagnostic code for the same admission was almost 8-fold higher 3 months post-CDI (hazard ratio (HR) = 7.56; 95% confidence interval (CI): 2.97–19.19) than for subjects without CDI and remained statistically significant until month 24 (HR = 1.47; 95% CI = 1.04–2.08). After CDI, patients remained at risk for gastrointestinal symptoms with CDI for up to two years. There is an important, long-term healthcare burden after CDI in this population.

## Introduction

*Clostridium difficile* infection (CDI) is one of the most urgent health concerns in the U.S. associated with antibiotic use [1], and the threat is magnified by the inappropriate overuse of antibiotics. Antibiotic exposure increases the risk of CDI for at least 3 months [2,3]. The risk of serious complications, including recurrent CDI (rCDI) and death, are high for 3–6 months after CDI particularly in patients who are older or have comorbid illness [4–7].

The impact of CDI in those high-risk patients is well documented. However, less is known about the course of a first episode of CDI in healthy nonelderly patients. We sought to explore how this episode of CDI affects future gastrointestinal (GI) symptomatology, including subsequent diagnoses of CDI, in a cohort of patients with a low prevalence of comorbid conditions. Because antibiotics and CDI can have a persistent effect on the gut microbiome [8–10] and changes to the gut microbiome have been associated with an increased risk for CDI, chronic GI disorders, obesity, and non-*C*. *difficile* enteric infections [11], we examined the immediate and long-term burden on any GI or subsequent CDI diagnosis. The objective of the study was to investigate if documented GI symptomatology returned to baseline in the 24 to 36 months after CDI in a nonelderly population.

## Methods

### Data source and sample

This study utilized administrative insurance claims data for hospital admissions contained in the Truven Health Marketscan Commercial Claims and Encounters database of private-sector health information of approximately 100 payers throughout the country [12]. This database represents approximately 50 million covered lives (annually) for employed subscribers younger than 65 years and their dependents. We performed a retrospective cohort analysis of hospital admissions. The claims data is fully anonymized prior to any access for analyses. The Boston University Medical Center Institutional Review Board classified this study as exempt from review.

Subjects <65 and > = 18 years of age and their adult dependents were included in the study sample if they met the following criteria: at least one inpatient claim for a *C*. *difficile* diagnosis (ICD-9 code: 008.45) between January 1, 2011 and December 31, 2011 (the calendar date of the first claim was set as time zero and labeled the *index date*); 18 years or older on the index date; at least 12 months of continuous pre-index enrollment (designated *baseline period*); between 24–36 months of continuous enrollment information after the index date (designated *follow-up period*) until December 31, 2013. Follow-up during 2011 depended on the index date of the *C*. *difficile* diagnosis and ranged from 0 to 12 months. All patients had complete data for 2012 and 2013. All subjects were alive at the end of their follow-up period. Subjects were excluded if they had any claim of a *C*. *difficile* diagnosis during the baseline period because the purpose of the study was to examine how their GI-health trajectory changed after a first episode of CDI.

### Measurement of gastrointestinal diagnoses, *C*. *difficile* diagnoses and covariates

All-cause hospital admissions during the follow-up period were examined for the following GI-related diagnoses (GIDx) abdominal pain; diarrhea; constipation; irritable bowel syndrome; weight loss; unspecified noninfectious gastroenteritis and colitis; nausea and vomiting (see S1 Table for relevant ICD-9 codes used to identify those diagnoses in the claims data). We counted the number of codes for GIDx per admission and computed the average events in three-month windows. Including baseline and follow-up periods, we analyzed sixteen, 3-month periods. We then dichotomized the summary count of GIDx to create a separate binary outcome: at least one GI diagnosis versus no diagnosis during a hospital admission. This version was generated to allow the calculation of hazard ratios (HRs) using a survival analysis framework.

At follow-up, we analyzed whether a *C*. *difficile* diagnostic ICD-9 code was included in hospital admissions claims after the index admission. In order to use it as a time-dependent predictor, first we counted the number of admissions with a *C*. *difficile* ICD-9 code (after the index date) and computed 3-month averages. Then, we created a dichotomous version for each 3-month interval; i.e., at least one *C*. *difficile* diagnosis vs. no *C*. *difficile* diagnosis per unique patient. Our main interest was in the association between GI diagnoses and the exposure (follow-up time). The152-155) other covariates measured were age, gender, U.S census regions (West, Midwest, South, and Northeast) and area of residence (urban, rural) at the index date. Census regions and area of residence were included in our analysis because published data demonstrates an association with those factors and different antibiotic usage patterns; different usage patterns might impact the risk for CDI [13]. We applied the comprehensive Elixhauser index [14] to control for pre-existing medical conditions of the heterogeneous populations within our sample and as a marker of severity of comorbid disease. Clinical variables were measured at baseline.

### Statistical analyses

The 12 months of each subject’s enrollment prior to the index date for a CDI served as the reference period for the analyses of that subject’s post-CDI time periods. We calculated the 3-month unadjusted incidence rates (per 10,000 person-years) throughout baseline and follow-up. We used Cox proportional hazards models to estimate the HR and 95% confidence intervals (CI) of GIDx adjusted for a pre-specified set of baseline demographic and clinical factors. The Cox regression provides risk estimates over time. It also allows the incorporation of time-dependent covariates (age, Elixhauser index) and can be adjusted to account for periods of time in which an individual is not at risk of an event. Finally, it can be implemented to control for the repeated events nature of the sample and allows combining time-dependent and discrete data in the same model.

Before running the model, we evaluated several assumptions. First, we assessed *ad-hoc* the dependency of measurements within individuals (residual test of within-subject correlation) which indicated that the data were correlated (p = 0.015). Due to this correlation, indicating diagnoses were not independent over time, particularly repeated diagnoses over multiple hospital admissions, we calculated robust standard errors to account for the dependence among GIDx. Then, we assessed the assumption of proportional hazards by testing the interaction between the time-dependent covariates and the natural logarithm of follow-up time (i.e., that the risk of a GI diagnosis was proportional across time). The assumption was met (p = 0.341). We specified a common origin for all events and calculated the hazard as a function of time since the index date. The final Cox model used the fixed effects partial likelihood method, which allows each individual to have their own hazard function and tolerates different subgroups having different baseline hazard functions, while constraining the coefficients to be the same across subgroups [15]. Interactions were examined for follow-up time for each of the remaining covariates and reported if they were significant or clinically relevant. After exploring the interactions between the exposure and the covariates, the effect of a CDI diagnosis on GIDx changed depending on time of follow-up (p < .001). Therefore, we reported the interaction estimates between CDI and 3-month averages. Collinearity was examined by assessing the Variance Inflation Factor (VIF) and not found among all covariates; no VIF exceeded a cut-off of 10 in our analyses. HRs were computed from the Cox models and were interpreted as relative risks or percentage increases or decreases associated with the covariate. All analyses were performed with SAS, version 9.4. A p<0.05 was considered statistically significant.

## Results

**Fig 1:**
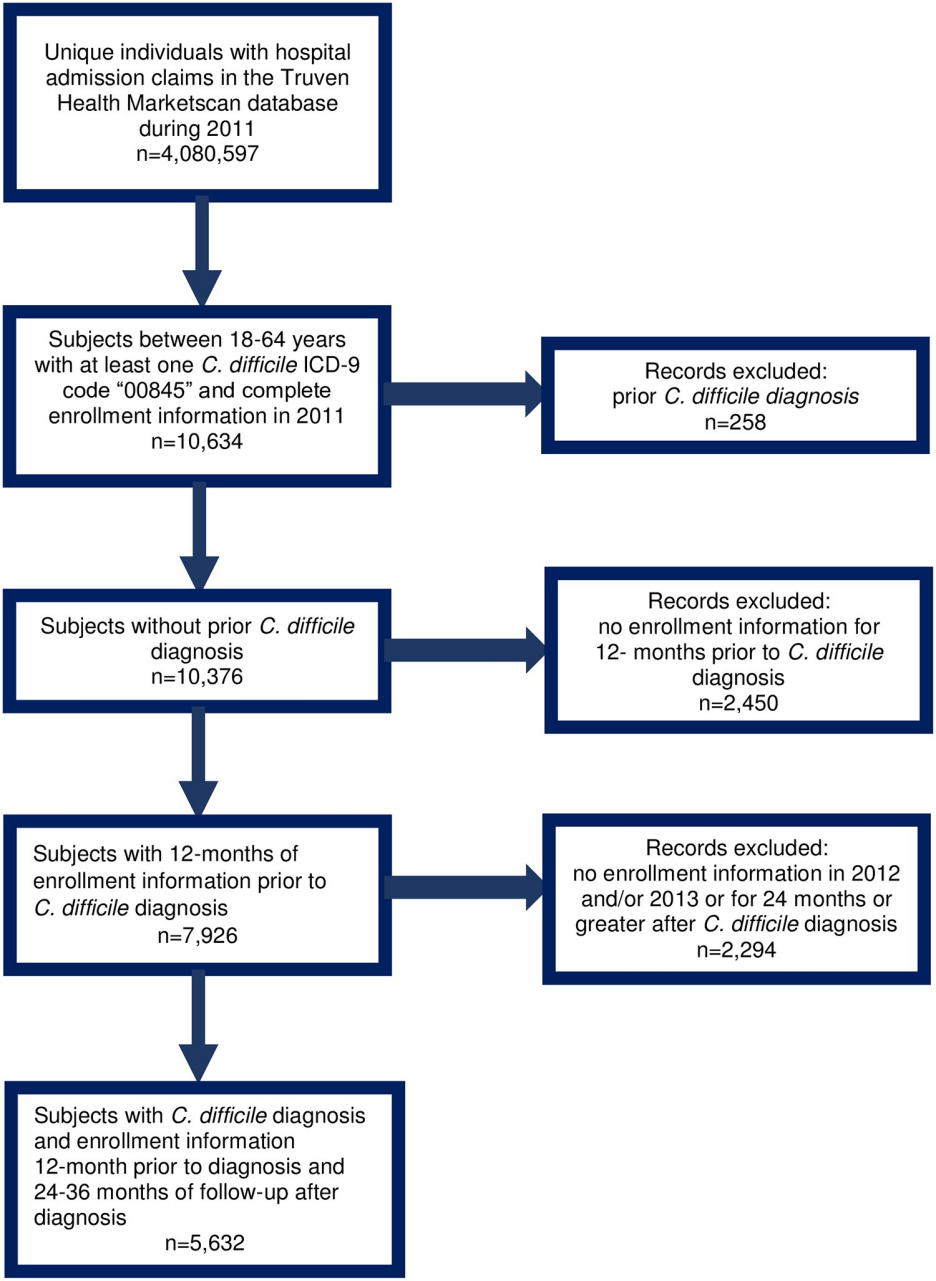
Selection criteria flowchart.

There were 4,080,597 unique individuals aged one to 64 years admitted to the hospital in 2011; 12,025 had at least one *C*.*difficile* diagnosis and complete enrollment information for 2011 (12,025/ 4,080,597 = 0.29%) and of those, 10,634 persons were aged 18–64 years of age (Fig 1). After applying the inclusion and exclusion criteria, a total of 5,632 persons had complete information (12 months of baseline and at least 24 months of uninterrupted follow-up enrollment information). Table 1 reports demographic characteristics. On average, patients were 47 years old, the majority were female, over 85% lived in urban areas, and about one third resided in the Southern region of the U.S.

**Table 1:** Study sample baseline characteristics at index admission for *Clostridium difficile* compared with all Truven hospital admission claims in 2011.

Characteristic	Study sample (n = 5,632)	Truven hospital admission claims
Age, mean years (SD)*	47.49 (16.48)	41.38 (16.55)
Gender, No. (%)		
Male	2,128 (37.8)	26,323,795 (42.76)
Female	3,504 (62.2)	35,234,987 (57.24)
Region, No. (%)		
Midwest	1,698 (31.0)	17,043,470 (27.69)
Northeast	996 (18.2)	6,756,513 (10.98)
West	920 (16.8)	7,137,796 (11.60)
South	1,863 (34.0)	30,501,806 (49.55)
Urban Residence, No. (%)	4,810 (85.4)	48,440,557 (78.69)

*SD denotes standard deviation.

**Fig 2:**
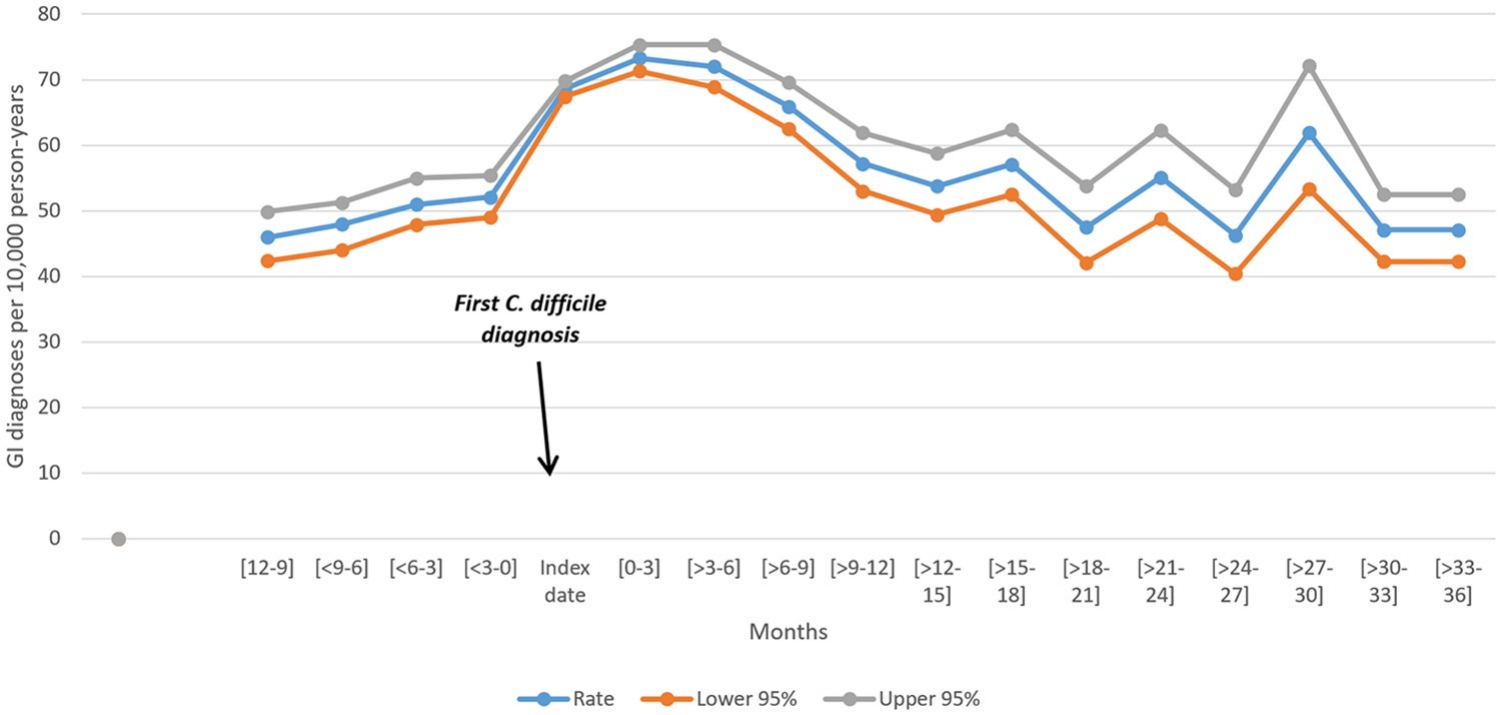
Unadjusted rate per 10,000 person-years for gastrointestinal diagnoses before and after first *Clostridium difficile* diagnosis.

The rates of GIDx per 10,000 person-years at baseline and follow-up before adjustments are presented in Fig 2 (see also S2 Table). The average rate of GIDx during hospital admission was elevated for the first year of follow up (69.9 per 10,000 person-years) compared to the 12-month baseline period (51.5 per 10,000 person-years). The second and third year of follow-up had average rates of 54 and 52.5 GIDx per 10,000 person-years, respectively, compared to baseline.

**Fig 3:**
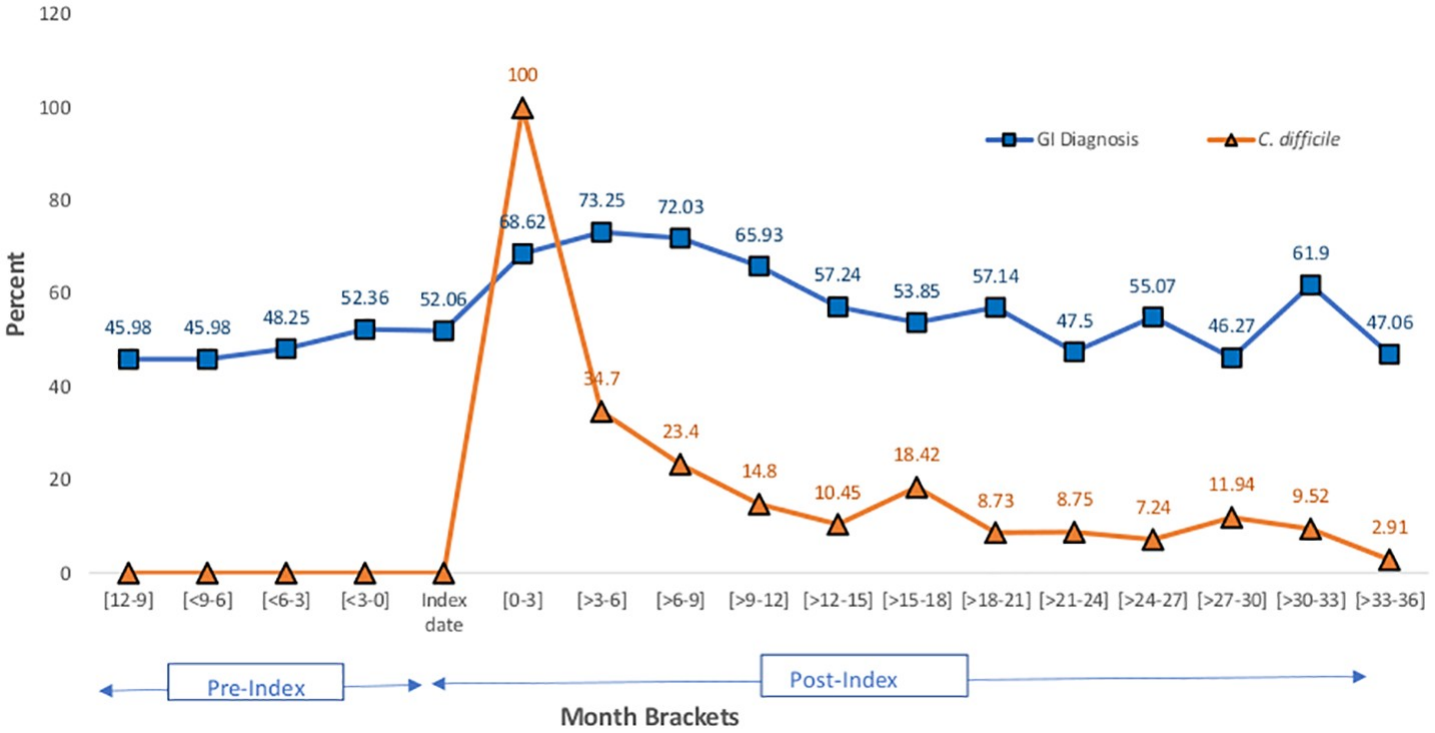
Prevalence of gastrointestinal (GI) diagnoses and *Clostridium difficile* diagnosis per person-admission at baseline and follow-up.

Fig 3 shows the effect of the first diagnosis of *C*. *difficile* on the proportion of GI and CDI diagnoses at follow-up (see also S3 Table). CDI was analyzed separately from GIDx and was not included as a diagnostic code in the GIDx data. The level of GI diagnostic codes during hospital admissions appears stable at baseline. At follow up, we observed an increased proportion of hospital admissions with GI diagnostic codes that remained elevated for at least 21 months compared with baseline. In a separate analysis, almost one third of admissions recorded a CDI diagnostic code after three months of follow up. Between months 6 and 15 of follow-up, a downward trend of CDI diagnoses were observed and plateaued, on average, at 10.7% between months 15 and 27. For patients with GIDx during hospitalization, the average number of specific GIDx codes per admission increased from 4.19 (SD 8.14) at the time of the index CDI to 5.5 (SD 9.75), 7.99 (SD 13.88), and 7.42 (SD 12.62) codes at one, two, and three years of follow-up, respectively.

After adjusting for baseline characteristics, HRs of GI diagnoses before and after the CDI index date compared to patients without GI diagnoses are shown in Table 2. There was a statistically significant interaction between follow-up time to GIDx and a CDI diagnostic code for that admission. At baseline, the presence of GIDx was not significantly different among the cohort and did not predict future GIDx during the follow-up period (HR = 1.04; 95% CI = 0.99–1.09). However, the risk of GI diagnostic codes for subjects with a CDI diagnostic code during the same admission at three months of follow up was almost 8-fold higher than that of subjects without a CDI diagnostic code (HR = 7.56; 95% CI: 2.97–19.19). The increased risk declined with time but remained significant until month 24 (HR = 1.46; 95% CI = 1.05–2.03). The Northeast and West had decreased risk of GIDx compared to the South. Gender was not associated with a higher risk of GIDx. Higher comorbidities as measured by the Elixhauser index increased the risk of GIDx (HR = 1.32; 95% CI: 1.12–1.56). As age increased, there was a small but significant association with fewer GIDx (HR = 0.997; 95% CI: 0.994–0.999) of uncertain clinical relevance.

**Table 2:** Risk estimates of gastrointestinal (GI) diagnoses after an index case of *Clostridium difficile*.

Variable	Unadjusted Hazard Ratio (95% CI)		Adjusted Hazard Ratio (95% CI)	
CDI* interaction with time (after index date^†^)				
3months	7.92	3.14–19.97	7.56	2.97–19.19
6 months	6.23	2.92–13.28	5.98	2.78–12.86
9 months	4.90	2.71–8.85	4.73	2.59–8.64
12 months	3.85	2.51–5.92	3.74	2.40–5.83
15 months	3.03	2.28–4.02	2.96	2.19–3.89
18 months	2.38	1.98–2.86	2.34	1.93–2.84
21 months	1.87	1.51–2.33	1.85	1.50–2.28
24 months	1.47	1.04–2.08	1.46	1.05–2.03
27 months	1.16	0.70–1.91	1.16	0.71–1.87
30 months	0.91	0.47–1.77	0.91	0.48–1.74
33 months	0.72	0.31–1.65	0.72	0.32–1.62
36 months	0.56	0.20–1.53	0.57	0.21–1.51
Age^‡^	0.998	0.996–1.00	0.997	0.994–0.999
Gender (Male)	1.07	0.99–1.15	1.01	0.93–1.11
GI diagnoses before index date^†^	0.52	0.49–0.55	1.04	0.99–1.09
Elixhauser index ^‡^	1.47	1.30–1.66	1.32	1.12–1.56
Region				
Midwest	1.03	0.94–1.13	0.99	0.90–1.09
Northeast	0.86	0.77–0.97	0.87	0.77–0.99
West	0.87	0.78–0.98	0.82	0.71–0.95
South	Reference			

*CDI: *Clostridium difficile* diagnosis;

^†^Index date = Calendar date of the first *Clostridium difficile d*iagnosis;

^‡^Time-dependent covariates

## Discussion and conclusions

*C*. *difficile* infection poses a major public health burden resulting in serious, and at times fatal, outcomes for those affected. This issue has taken on greater importance because of the increased attention on the inappropriate use of antibiotics in medicine. The morbidity and mortality of CDI has been well described in high-risk populations–the elderly and patients with serious underlying conditions. However, the total healthcare impact may be underestimated because outcomes of CDI in a nonelderly population (<65 years of age) with low rates of comorbid conditions has not been well studied.

Unlike most published data, our cohort sample, an employed, middle-class population and their dependents, did not include any subjects over 65 years of age. It is known that outcomes are worse in older patients [16]. In a population-based cohort study of US adults aged 65 years or older, patients with CDI had almost twice the mortality and healthcare costs compared with a propensity-matched sample of patients without CDI [16]. In another study, severe colitis and treatment failure were significantly more common in CDI patients over 65 years of age than those who are younger [17]. Little is known about the healthcare burden in nonelderly patients.

In addition, the overall database used for our study reflects a population with lower comorbidities overall than other large databases available for analysis including low rates of those comorbid risk factors associated with CDI [18]. It has been extensively documented that patients with comorbid conditions such as cancer or chronic renal failure have progressively worse outcomes after CDI but there are sparse data in healthier cohorts [5,7,19].

Consistent with prior research that recurrent disease and hospital readmissions are frequent for 3–6 months after a CDI diagnosis [4], the highest risk of CDI and GIDx in our cohort was also within the first 3–6 months of post-CDI follow-up. Published data with follow-up beyond six months primarily focus on mortality. For example, a study by Doh et al found two-year all-cause mortality after CDI was 32.5% [20]. Kuntz et al found higher levels of healthcare utilization and greater 1-year mortality in patients with rCDI than in those who never had CDI or who never had rCDI [21]. Reacher et al demonstrated excess mortality of ~50% within the first year after CDI [22]. Hensgens et al demonstrated the *C*. *difficile*-related mortality risk was highest in the first 30 days after infection, but remained elevated at one year [23]. All subjects in our cohort were alive at the end of the study.

We were only able to identify one published study with long-term follow-up that focused on a healthy, young cohort. Gutierrez et al performed a retrospective cohort study of GI sequelae after CDI in active-duty military personnel with a median age of 27 years and median follow-up of three years [24]. They found CDI was increasing in this nonelderly healthy population and was associated with clinically relevant GI functional disorders. Our population is more generalizable than this active-duty military cohort. For example, patients who were over 40 years of age or female were disproportionately absent from their sample. They used a baseline period prior to the index CDI of only 12 weeks. Our 12-month baseline period is less likely to miss earlier diagnoses of CDI that might have impacted outcomes. Finally, they did not include CDI as a factor during the follow-up period. Other studies have retrospectively examined the risk for post-infectious irritable bowel disease 3 to 9 months after CDI; rates have varied from 4 to 25% [25–27]. Unlike our study, the sample sizes were small (23–315 subjects) and in two of these studies, follow-up was less than one year [25,26]. In the study by Jackson et al, only patients returning to care for a possible episode of rCDI were included and 45% of that cohort had no additional follow-up [27]. The largest study by Wadhwa et al was a survey with only a 46% response rate and a high risk for bias [26].

Our results suggest that CDI is associated with significant effects on the overall GI symptomatology in our nonelderly cohort of patients less than 65 years of age. We found the number of GI diagnoses during hospital admissions did not return to baseline for almost two years of follow-up and there was an increased risk of CDI with GIDx for 24 months after the index infection.

A strength of our study is including and linking GI diagnoses with CDI in our analyses. CDI is associated with multiple risk factors [28] that perturb the gut homeostasis–antibiotics, proton-pump inhibitors, obesity and inflammatory bowel disease [29–35]. Continued disease may not be due exclusively to CDI relapse or reinfection. We analyzed the risk of GI diagnoses over time, as well as the prevalence and number of GI symptoms during any hospital admission. GI diagnoses peaked after the baseline episode of CDI and then subsided but did not return to baseline during most of the follow-up period. Controlling for other factors, the risk of GI diagnoses in subjects with a CDI diagnosis during the same hospitalization remained elevated for two years. Subjects admitted with GI complaints during the second and third year of follow-up had high numbers of specific GI diagnostic codes even as the diagnosis of CDI declined dramatically. Thus, even in patients not previously identified as high risk for poor outcomes, there is a long-term health burden after CDI.

This study has several limitations. First, our data includes only individuals with employer-based insurance and their beneficiaries and may not be generalizable to the uninsured, underinsured, and those who rely solely on state and federal healthcare coverage like Medicaid. Further, these study results accounted for age, gender, and geographic location to calculate the hazard estimates, and may not be generalizable in terms of race or education. In addition, inclusion required subjects to be within the Truven database for at least 3–4 years (a baseline year and 2–3 years of follow-up after an index case of CDI). Those with stable private insurance for that time frame may differ from the general Truven population. Second, all CDI and GI diagnoses and comorbid conditions were based on claims data using ICD-9 codes. Surveillance for CDI by ICD-9 code has been shown to have poor sensitivity when compared with laboratory diagnoses [36,37]. Although there is no specific data for the Truven database, studies have demonstrated the correlation between ICD-9 codes and test data to be strong (e.g., a specificity of 99.7% in one study [37]). We included only those subjects without any evidence of a *C*. *difficile* diagnostic code for at least 12 months prior to CDI which strengthens our confidence in the accuracy of the index diagnosis of CDI. We are less certain, however, that coding for CDI during any future hospitalizations reliably indicated active CDI. Codes for *C*. *difficile* may have been included because of a prior history, particularly a recent history. However, the number of admissions with CDI ICD-9 codes decreases over time despite the persistence of GI diagnostic codes, suggesting the code is not used indefinitely after an index case (see Fig 3 and S3 Table). In addition, our model assessed the risk of GI diagnoses as predicted by a CDI. By linking the two diagnoses, we increased the likelihood that CDI was clinically relevant during the admission.

Similarly, the claims data limits our ability to know whether all GI diagnoses received an ICD-9 code, particularly when more severe diagnoses were present. However, this would serve to underestimate GI symptomatology making our results more conservative and suggest that long-term GI conditions may have been more prevalent than our study can define.

A third limitation is the lack of information on other reasons for admission that might influence GI disease or further episodes of CDI. We also lack medication prescribing data because inpatient claims do not include drug information and all resources used during a hospitalization are reported in aggregate. Correlation of CDI ICD-9 codes with evidence of treatment for CDI would have strengthened our certainty that CDI was an active problem. In addition, data on antibiotics or medications linked to occurrence of CDI might have impacted the risk for long-term GI conditions and subsequent episodes of CDI. As half of the hospitalizations pre-CDI were associated with GI symptoms, medication history might also have helped to assess patients with pre-existing GI problems as a risk for poor CDI outcomes.

Fourth, although the database overall is a population with low comorbidities, we do not know how the comorbidities of the subjects in our cohort with CDI compares to the overall dataset or the prevalence of potential risk factors for poor outcomes after CDI. There may be a subset within our nonelderly cohort who had long-term GIDx after CDI because of known risk factors.

Finally, our analyses between 24 and 36 months of follow-up may be less reliable due to insufficient power during that time period. Analyses using more robust data beyond 24 months are needed for future work.

Despite the limitations of claims data, and uncertainties regarding the accuracy of the ICD-9 coding, our results suggest compelling preliminary evidence for long-term health impacts after CDI, specifically for persistent GI diagnoses and relapse or reinfection with CDI. Confirmation of these findings through more granular cohort studies with chart review rich in clinical data would be a productive and important focus for future research. Identification of risk factors predictive for readmissions and rCDI in nonelderly cohorts would help inform future strategies to reduce the burden of CDI for these patients and the high costs to the healthcare system [17,38,39]. Patients at high risk for poor outcomes might benefit from earlier, more aggressive strategies to restore or maintain the microbiome [40], such as with fecal microbiota transplantation [41]. Additional research can more thoroughly examine recurrent CDI versus reinfection, primary and secondary diagnoses associated with each hospital admission and the impact of prescription medications.

In conclusion, patients with an episode of CDI remained at risk for GI symptoms with CDI for up to two years. Our study demonstrates that long-term impacts are important and CDI poses a greater burden to public health than previously demonstrated, even in this relatively healthy nonelderly cohort.

## Supporting information

S1 Table *Clostridium difficile* and gastrointestinal diagnoses ICD-9 codes. (DOCX)

S2 Table Unadjusted rate per 10,000 person-years for gastrointestinal diagnoses before and after first *Clostridium difficile* diagnosis. (DOCX)

S3 Table Prevalence of gastrointestinal (GI) diagnoses and *Clostridium difficile* diagnosis per person-admission at baseline and follow-up. (DOCX)
