# Supplementary material for: Retrospective analysis of long-term gastrointestinal symptoms after Clostridium difficile infection in a nonelderly cohort
Source: PLoS One. 2018 Dec 17;13(12):e0209152. doi: 10.1371/journal.pone.0209152 (PMC6296708; doi:10.1371/journal.pone.0209152)
Supplement: S1 Table — (DOCX) [file pone.0209152.s001.docx]

| **Gastrointestinal Diagnosis** | **ICD-9 CODES** |
| --- | --- |
| *Clostridium Difficile* | 00845 |
| Abdominal Pain | 78900,78901,78902,78903,78904,78905,78906,78907,78909 |
| Diarrhea | 78791, 5645 |
| Constipation | 564, 5640,5601,56402,56409 |
| Irritable Bowel Syndrome | 5641,7810 |
| unspecified noninfectious gastroenteritis and colitis | 5589,0090, 0091 |
| Weight Loss | 78321 |
| Failure to thrive | 7837 |
| Anorexia | 7830 |
| Nausea and vomiting | 78702, 78701, 78703 |
| Dyspepsia | 5368 |

**S1 Table: *Clostridium difficile* and gastrointestinal diagnoses ICD-9 Codes**
