# Supplementary material for: Retrospective analysis of long-term gastrointestinal symptoms after Clostridium difficile infection in a nonelderly cohort
Source: PLoS One. 2018 Dec 17;13(12):e0209152. doi: 10.1371/journal.pone.0209152 (PMC6296708; doi:10.1371/journal.pone.0209152)
Supplement: S2 Table — (DOCX) [file pone.0209152.s002.docx]

**S2 Table: Unadjusted rate per 10,000 person-years for gastrointestinal diagnoses before and after first *Clostridium difficile* diagnosis**

| **Time Period** | **Rate** | **Lower 95%** | **Upper 95%** |
| --- | --- | --- | --- |
| 12 months Pre-index | 46.0 | 42.4 | 49.9 |
| 9 months Pre-index | 48.0 | 44.0 | 51.3 |
| 6 months Pre-index | 51.0 | 47.9 | 55.0 |
| 3 months Pre-index | 52.1 | 49.0 | 55.4 |
| Index date | 68.6 | 67.4 | 69.8 |
| 3 months Post-index | 73.3 | 71.3 | 75.3 |
| 6 months Post-index | 72.0 | 68.9 | 75.3 |
| 9 months Post-index | 65.9 | 62.5 | 69.6 |
| 12 months Post-index | 57.2 | 53.0 | 61.9 |
| 15 months Post-index | 53.8 | 49.4 | 58.8 |
| 18 months Post-index | 57.1 | 52.5 | 62.4 |
| 21 months Post-index | 47.5 | 42.1 | 53.8 |
| 24 months Post-index | 55.1 | 48.8 | 62.3 |
| 27 months Post-index | 46.3 | 40.4 | 53.2 |
| 30 months Post-index | 61.9 | 53.3 | 72.1 |
| 33 months Post-index | 47.1 | 42.3 | 52.5 |
| 36 months Post-index | 49.3 | 44.1 | 54.7 |
