# Supplementary material for: Retrospective analysis of long-term gastrointestinal symptoms after Clostridium difficile infection in a nonelderly cohort
Source: PLoS One. 2018 Dec 17;13(12):e0209152. doi: 10.1371/journal.pone.0209152 (PMC6296708; doi:10.1371/journal.pone.0209152)
Supplement: S3 Table — (DOCX) [file pone.0209152.s003.docx]

**S3 Table: Prevalence of gastrointestinal (GI) diagnoses and *Clostridium difficile* diagnosis per person-admission at baseline and follow-up**

| Months | GI Diagnoses No. (%) | *C. difficile* No. (%) | Denominator:  No. inpatient admissions* |
| --- | --- | --- | --- |
| 12 months pre-index | 357 (45.98) | - | 777 |
| 9 months pre-index | 496 (48.25) | - | 1,028 |
| 6 months pre-index | 890 (52.36) | - | 1,700 |
| 3 months pre-index | 590 (52.06) | - | 1,134 |
| Index date | 3,864(68.62) | 5,632 (100) | 5,632 |
| 3 months post-index | 1,892(73.25) | 898 (34.74) | 2,584 |
| 6 months post-index | 875 (72.03) | 285 (23.44) | 1,215 |
| 9 months post-index | 633 (65.93) | 142 (14.81) | 961 |
| 12 months post-index | 383 (57.24) | 70 (10.45) | 669 |
| 15 months post-index | 313 (53.85) | 107 (18.42) | 582 |
| 18 months post-index | 303 (57.14) | 46 ( 8.73) | 532 |
| 21 months post-index | 198 (47.50) | 37 ( 8.75) | 418 |
| 24 months post-index | 214 (55.07) | 28 ( 7.24) | 390 |
| 27 months post-index | 164 (46.27) | 43 (11.94) | 356 |
| 30 months post-index | 178 (61.9) | 27 ( 9.52) | 287 |
| 33 months post-index | 222 (47.06) | 14 ( 2.91) | 472 |
| 36 months post-index | 140 (49.18) | 9 ( 3.27) | 285 |

*The full cohort of 5,632 patients was followed for at least 24 months.
